# Intercellular communication between FAP+ fibroblasts and SPP1+ macrophages in prostate cancer via multi-omics

**DOI:** 10.3389/fimmu.2025.1560998

**Published:** 2025-05-14

**Authors:** Tingting Wu, Xinyu Li, Fei Zheng, Hanchao Liu, Yang Yu

**Affiliations:** ^1^ Department of General Surgery, Shenzhen Qianhai Taikang Hospital, Shenzhen, China; ^2^ Department of Urology, The First Affiliated Hospital of Wenzhou Medical University, Wenzhou, Zhejiang, China; ^3^ Department of Obstetrics and Gynecology, Shengjing Hospital of China Medical University, Shenyang, China; ^4^ Department of Andrology and Urology, Sir Run Shaw Hospital, affiliated with the Zhejiang University School of Medicine, Hangzhou, Zhejiang, China; ^5^ Department of General Surgery, Chifeng Hospital, Chifeng, Inner Mongolia, China

**Keywords:** prostate cancer, tumor microenvironment, cellular crosstalk, single-cell RNA sequencing, spatial transcriptomics, FAP+ fibroblasts, SPP1+ macrophages, immunotherapy

## Abstract

**Background:**

Prostate cancer (PCa) presents substantial heterogeneity and unpredictability in its progression. Despite therapeutic advancements, mortality from advanced PCa remains a significant challenge. Understanding the intercellular communication within the tumor microenvironment (TME) is critical for uncovering mechanisms driving tumorigenesis and identifying novel therapeutic targets.

**Methods:**

We employed an integrative approach combining bulk RNA sequencing, single-cell RNA sequencing (scRNA-seq), and spatial transcriptomics to investigate interactions between FAP+ fibroblasts and tumor-associated macrophages in PCa. Key findings were validated using immunohistochemical and immunofluorescence staining techniques.

**Results:**

Analysis of 23,519 scRNA-seq data from 23 prostate samples revealed a pronounced accumulation of FAP+ fibroblasts in tumor tissues. Spatial transcriptomics and bulk RNA sequencing demonstrated strong associations between FAP+ fibroblasts and SPP1+ macrophages. Notably, tumor-specific intercellular signaling pathways, such as CSF1/CSF1R and CXCL/ACKR1, were identified, highlighting their potential role in fostering an immunosuppressive TME.

**Conclusion:**

Our findings unveil a distinct pattern of crosstalk between FAP+ fibroblasts and SPP1+ macrophages in PCa, shedding light on potential therapeutic targets for advanced PCa.

## 1 Background

Prostate cancer(PCa) constitutes one of most prevailing malignancies in the male urinary system which prominently contributes to high mortality rates in old men around the world (1). Nowadays, the treatment strategies of prostate cancer mainly include surgery, chemotherapy, radiotherapy, endocrine therapy, immunotherapy and so on (2). However, due to the concealed biological behavior of PCa and the absence of valid early screening methods, most PCa patients are diagnosed at advanced stage, and more than 30% of patients experience biochemical recurrence after treatment (3), severely impacting the survival time and quality of life of PCa patients (4). Therefore, there is an increasing demand for biomarkers and therapeutic targets that can accurately predict the prognosis of PCa and guide treatment decisions, ultimately improving the prognosis of PCa.

Recent years, the development of novel nanomaterials and nanoparticles has provided a promising carrier for tumor drug delivery system (5–7). However, previous treatments targeting cancer cells have encountered various challenges like tumor metastasis, recurrence, and medicine resistance (8). Therefore, concept of the tumor microenvironment (TME) is introduced to improve treatment strategy of PCa. The TME composed of cancer-associated fibroblasts (CAFs), tumor-associated macrophages (TAMs), endothelial cells, extracellular matrix and secreted signaling molecules, plays a pivotal role in modulating cancer progression, immune escape, and therapeutic response (9, 10). Recent advances in single-cell RNA sequencing (scRNA-seq) and spatial transcriptomics (ST) have enabled a deeper understanding of cellular heterogeneity and spatial dynamics within the TME (11–15).

The TME in PCa is highly dynamic and exhibits profound variability in cell composition and spatial interactions. For instance, CAFs have been reported to induce regulatory T cells and promote an immunosuppressive niche, while TAMs could facilitate angiogenesis and lead to cancer resistance to chemotherapy (10). These cellular components establish a reciprocal communication network that drives lineage plasticity, immune evasion, and castration resistance (16–21).

However, scRNA-seq lose the spatial information of cells, and it is difficult to accurately reveal the complex cellular interactions within the TME solely relying on scRNA-seq (22). The emergence of ST allows us to explore gene expression, cell differentiation, and cell-cell communications among cells without losing their spatial information (23, 24). Therefore, the integrational analysis of scRNA-seq and ST not only enables efficient exploration of the functions and interactions of different cellular subgroups within the TME but also allows for the investigation of spatial heterogeneity within tumor tissues, which greatly enhances our understanding of the TME (25).

The interactions between CAFs and TAMs have garnered extensive attention (19, 26). However, due to the complexity of TME, the sub-classification of these cells and their specific interactions remain unclear, necessitating further exploration and research. In this study, we focused on the interactions between FAP+ fibroblasts and SPP1+ macrophages within the PCa TME, combining scRNA-seq and ST with functional network analysis to reveal their spatial colocalization and molecular crosstalk. Our findings provide novel insights into the landscape of intercellular communication underlying PCa progression.

## Methods

### Data sources

The scRNA-seq data were downloaded from the GEO database, with associated numbers of GSE166782 (4 samples of normal prostate tissue) and GSE176031 (19 samples of PCa tissues). The detailed information of the scRNA-seq samples is shown in **Supplementary Figure S2 (**, 27). The ST data were downloaded from the datasets section of the official website of 10X Genomics (https://www.10xgenomics.com/) by searching for “prostate”. The bulk RNA transcriptomics data of PCa were downloaded from the PRAD cohort of the TCGA database at the website: https://xena.ucsc.edu/, and from the GEO database with associated numbers of GSE70768 (125 tumour samples from patients with PCa) and GSE70769 (103 tumour tissue samples from men with PCa) (28). The overall design of this research was also shown in **Supplementary Figure S1**.

### scRNA-seq analysis

The scRNA-seq data processing was conducted using the Seurat package (v4.0.2) in R (v4.0.5). Metadata row names were updated with sample information, followed by the merging of 23 selected samples into a single Seurat object. This object then underwent filtration and normalization, retaining cells with over 800 genes expressed and less than 25% mitochondrial genome reads. The integrated Seurat objects were then scaled and analyzed via principal component analysis (PCA) to reduce the dimensionality of the data. The first 25 principal components were then utilized to construct a K-nearest neighbors (KNN) graph, refining edge weights between cells. Batch effects were mitigated using the harmony method (v0.1.0), facilitating the integration of Seurat objects into a unified dataset. Calculation of UMAP was carried out on this dataset, focusing on the top 3000 variable genes and employing the first 25 principal components. Cell clustering was performed via the FindClusters function with a resolution of 0.1, resulting in the identification of 9 distinct clusters on the basis of local neighborhoods. These clusters were subsequently annotated via established marker genes. For further subcluster analysis in TAMs and CAFs, the first 20 PCs were employed for nonlinear dimensionality reduction via UMAP. Clustering analysis was then repeated which led to the identification of 4 subclusters of CAFs and 4 subclusters of TAMs.

### Differential-expression analysis and cell identification

We identified differentially expressed genes (DEGs) in cell clusters from scRNA-seq data by the program of FindAllMarkers. Genes exhibiting positive expression in over 25% of cells within any given cluster were chosen. For functional analysis of cell clusters, clusterProfiler package (v3.18.1) was adopted for GO enrichment analysis, with a significance threshold set at p < 0.05 and |avg_logFC| > 1.5.

### ST analysis

To process and visualize the ST data, seurat was utilized. The SCT method was employed for ST data normalization, and the integration of ST data was achieved through functions such as SelectIntegrationFeatures, PrepSCTIntegration, FindIntegrationAnchors, and IntegrateData. We then applied an unsupervised clustering approach to group similar ST spots. The annotation of cell populations was informed by hematoxylin and eosin (HE) stained slices and the highly variable genes across clusters. For calculating cell-specific signature scores from scRNA-seq, we employed five methods: AUCell, UCell, singscore, ssGSEA, and AddModuleScore. Visualization of cell expression levels in ST data was conducted using SpatialDimPlot and SpatialFeaturePlot.

### Charaterization of cell−type infiltration

To assess the infiltration level of cell types in ST sequencing data and the TCGA cohort PRAD, we incorporated the top 20 DEGs from scRNA-seq along with wildly acknowledged immune cell gene sets. The ssGSEA algorithm was employed to calculate scores for angiogenesis and cytokine-interaction signatures in GSVA package (v1.38.2). For mapping the distribution of FAP+fibroblasts and SPP1+ macrophages in ST sections, we utilized the top 20 DEGs from each cluster, applying methods including AUCell algorithm, UCell algorithm, singscore algorithm, ssGSEA algorithm, and AddModuleScore function. Additionally, to examine the expression difference of FAP in tumors versus normal tissues across various cancer spicies, the online platform TIMER was employed (https://cistrome.shinyapps.io/timer/).

### Correlation analysis

To explore the relationships of the cell types we have allocated, correlation analysis and visualization were conducted using the R packages of ggstatsplot (v0.10.0) and corrplot (v0.92). Moreover, the online tool TIMER identified the correlations between immune infiltration and gene expression level within the PRAD cohorts. A p-value of less than 0.05 was defined statistically significant.

### Survival analysis

To examine the impact of specific cell populations on clinical outcomes, survival analyses were performed on the TCGA cohort PRAD, along with GSE70768 and GSE70769 datasets, using the Survival package (v3.2-10) and Survminer (v0.4.9). The infiltration levels of cell populations, based on the top 20 DEGs from scRNA-seq, were quantified with the ssGSEA algorithm. Patients were then stratified into groups with high or low levels of infiltration using the median value as the cutoff. Kaplan–Meier survival curve was generated with the survfit function, considering a p-value < 0.05 as statistically significant.

### Quantitatively analysis of cell communications

The CellChat package (v1.1.3) was employed to explore intercellular communications, particularly between cancer associated fibroblasts and tumor infiltrated myeloid cells, through the construction of a regulatory framework centered on ligand-receptor interactions. We excluded cell communications with less than 10 cells per specific subgroup. The netVisual function facilitated the visualization of interaction patterns. For calculate and display the contribution of single pair to the whole activity of the signaling pathways, the netAnalysis contribution function was used. The expression levels of each pair of ligands and receptors in specific signaling pathways across cell clusters were illustrated by violin plots via the PlotGeneExpression function. In determining the quantity of co-communication modes within the CellChat object, the non-negative matrix factorization (NMF) algorithm was applied, following the use of the identifyCommunicationPatterns function to discern primary signals and communication patterns among cell groups. Furthermore, network centrality scores were computed using the netAnalysis_Centrality function. The netAnalysis_signalingRole_network function was utilized for the graphical representation of network roles, aiding in the identification of predominant senders, receivers, mediators, and influencers within the inferred networks.

### Immunohistochemical staining

The immunohistochemical kit (Maixin Biotechnology, China) were used for the experiment. Firstly, human prostate tissue sections were dewaxed to water. The antigenic sites were exposed by microwave repair using sodium citrate antigenic repair solution. Subsequently, endogenous peroxidase blockers were applied to inactivate endogenous peroxidases. Following that, the slices were blocked in non-specific stain blockers for 30 min at 37°C. After adding primary antibodies (FAP, Rabbit, JA56-11, HUABIO, 1:1000), they were stored for incubation overnight at 4 °C. The following day, the sections were incubated with an HRP-conjugated secondary antibody for 20 min at room temperature. Diaminobenzidine (DAB, Maixin Biotechnology, China) was used as the chromogen and nuclei were stained with hematoxylin. The expression of FAP was observed under microscope, and the presence of brown-yellow or brown-yellow particles in the cells was the positive staining sign. The score was based on the percentage of positive stained cells and the intensity of the stain, and the two scores were multiplied to obtain the total score. According to the total score, the samples were divided into low expression group and high expression group.

### Immunofluorescence staining

Tissue was infiltrated with 0.5% Triton X-100 for 10 minutes. A non-specific stain blocker (Maixin Biotechnology, China) was added to the slices and incubated at room temperature for 10 minutes. FAP antibodies (Rabbit, JA56-11, HUABIO, 1:500) and SPP1 (Mouse, MH49021, Abmart, 1:300) were incubated overnight at 4°C. Anti-mouse fluorescent secondary antibody (SA00013-1, Proteintech, 1:500) and anti-rabbit fluorescent secondary antibody (SA00013-4, Proteintech, 1:300) were incubated at room temperature for 2 hours away from light. The cell nucleus was re-dyed with 4’,6-diamidino-2-phenylindole (DAPI) reagent (E-IR-R103, Elabscience, China) at room temperature, away from light, for 10 minutes. Finally, the sealing solution of anti-fluorescence quench agent was added to the slide, and the cover glass was covered. Taking pictures under a confocal microscope (LSM880, Zeiss, Jena, Germany).

## Results

### Construction of scRNA-seq atlas of PCa

To characterize the diverse the TME in PCa, we combined 2 scRNA-seq datasets previously published, encompassing 19 samples from tumor cores and 4 from normal prostate tissue, totaling 23,519 cells for analysis (**Supplementary Figure S2**). This dataset included 12,145 cells from tumor tissues and 11,374 from normal tissues.

**Figure 1:**
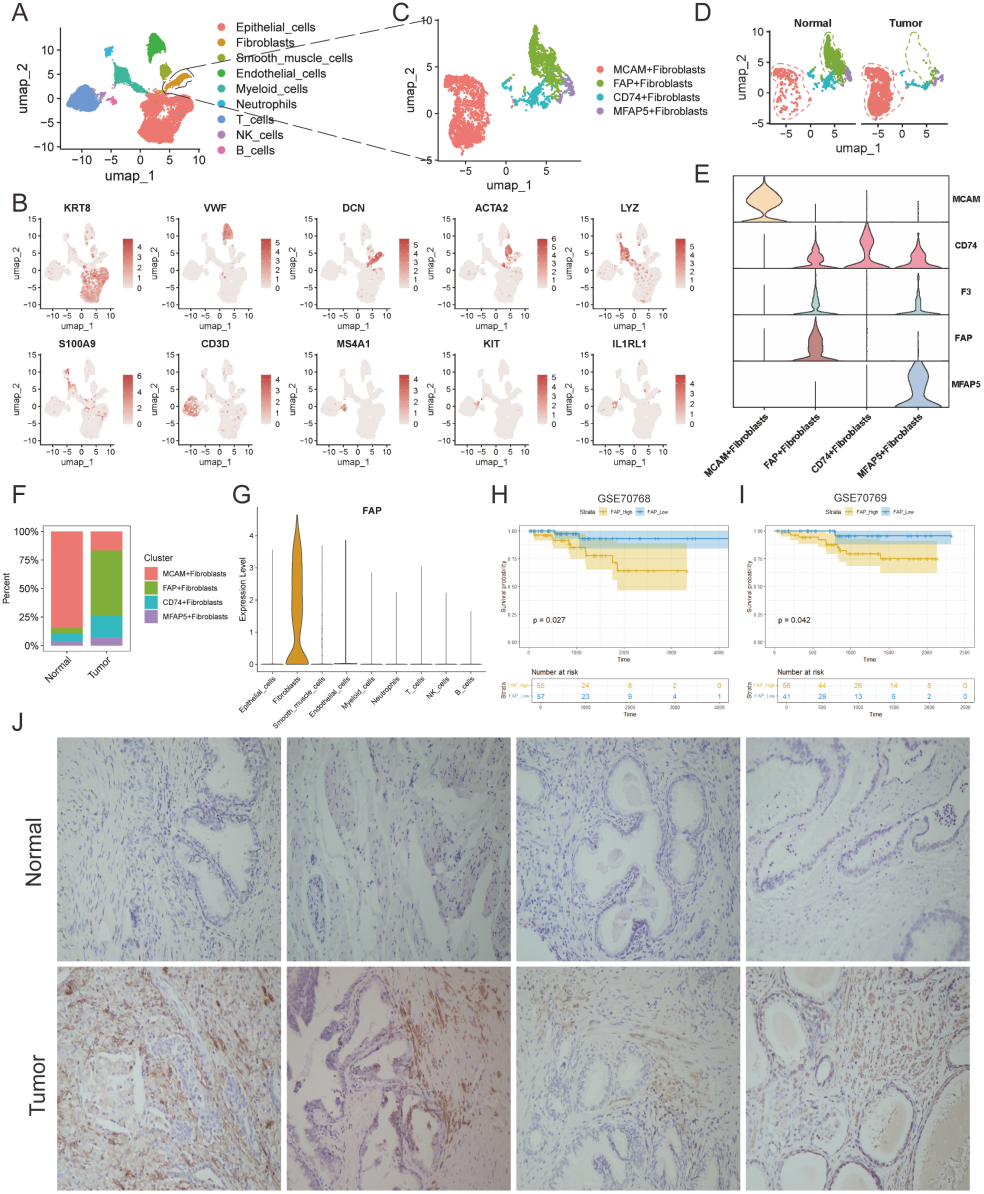
Tumor-specific microenvironment shaped the malignant characteristics of FAP + fibroblasts. **(A)** UMAP plots profiled the integrative analysis of scRNA-seq samples. **(B)** Stacked barplots showing the fractional composition of cell numbers for different clusters in normal and tumor tissues. **(C)** UMAP plot of scRNA-seq of fibroblasts in PCa. **(D)** UMAP plots of fibroblast subclusters faceted by tissue types. **(E)** Violin plot of fibroblast subclusters marker genes. **(F)** Bar plots show the percentage of each fibroblast subtypes in scRNA-seq. **(G)** Violin plot shows that the FAP is a fibroblast-specific signature in PCa. **(H, I)** Kaplan-Meier analysis of high-FAP group and low-FAP group about OS in in two PCa corhorts (GSE70768 and GSE70769). **(J)** IHC analysis show the existence of FAP + fibroblasts in tumor and normal tissues.

Based on established gene markers, we categorized the cell populations into 9 types: epithelial cells (12,747), identified by KRT8 and EPCAM expression; endothelial cells (2,144), expressing VWF and PECAM1; fibroblasts (855), marked by DCN, COL1A1, and COL1A2; myeloid cells (2,123), defined by LYZ; T cells (4,054), with CD3D and CD3E; neutrophils (397), expressing S100A9; B cells (271), identified by MS4A1; smooth muscle cells (824), marked by ACTA2; and NK cells (134), characterized by KIT expression (**Figures 1A, B**).

The distribution of these nine cell types varied across different tissues, with myeloid cells, B cells, neutrophils, NK cells, and T cells showing higher infiltration in tumor regions than in normal tissues, highlighting the heterogeneous microenvironment of PCa compared to normal prostate tissue (**Supplementary Figure S2**).

### Tumor microenvironment contributes to the malignant characteristics of cancer-associated FAP+ fibroblasts

CAFs have been identified as the most abundant stromal cells in TME and are recently recognized as occupying the highest tier in the hierarchy of cellular interactions (29). However, due to the complex heterogeneity of TME, the specific identity of these cells remains a challenge (30–32).To better elucidate the subtypes of fibroblasts in prostate tissue, generally acknowledged fibroblast markers, highly variable features, and functional enrichment analyses were integrated for subcluster annotation purposes (**Figures 1C, D**). Marker genes of FAP, MFAP5, CD74 and MCAM were used to identify the subclusters of fibroblasts(**Supplementary Figure S3**). Accordingly, the fibroblasts were classified into FAP+ fibroblasts, MFAP5+ fibroblasts, CD74+ fibroblasts, MCAM fibroblasts by the high expression degree of these marker genes (33) (**Figure 1E**).

Subsequently, we assessed the infiltration levels of each fibroblast subtype across different tissues, noting that FAP+ fibroblasts predominantly resided within tumor regions while MCAM+ fibroblasts were notably observed in normal region indicating their biological heterogeneity (**Figure 1F**). In addition, it is notable to find that FAP was specifically expressed in fibroblasts (**Figure 1G**). Next, we explore the pan-cancer differential expression of FAP comparing tumor tissues with normal prostate tissues using the online platform TIMER. Our findings indicated an upregulation of FAP in nearly all examined tumor types, with a notable increase in prostate adenocarcinoma (PRAD) (**Supplementary Figure S5**). Further analysis into the relationship between FAP and clinical prognosis revealed that elevated FAP expression related to poorer prognosis in PCa patients, which was also evidenced in two independent public cohorts (**Figures 1H, I**). In conclusion, we speculated that FAP+ fibroblasts may alter the biological properties of the TME through complex cell-to-cell interactions, thus resulting in the poor prognosis of PCa patients.

### Intercellular communications between FAP+ fibroblasts and TAMs revealed by multi-omics analyses

**Figure 2:**
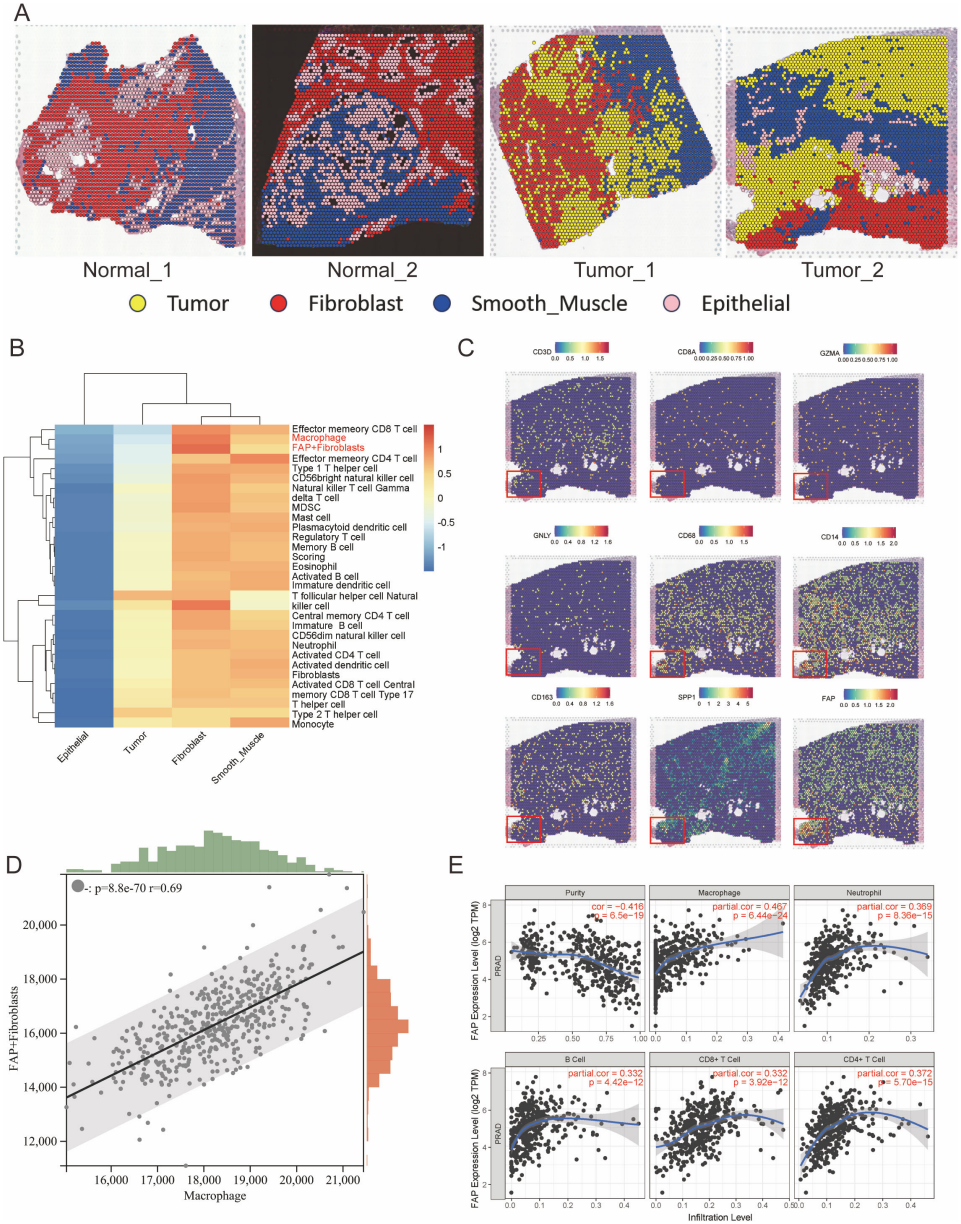
Spatial transcriptomics reveals co-localization of FAP^+^ fibroblasts and macrophages in prostate cancer tissues. **(A)** Spatial clustering of ST sections from normal and tumor prostate tissues. Spots were annotated into major cell types (Tumor, Fibroblast, Smooth Muscle, Epithelial) based on marker gene expression and histological features. **(B)** Heatmap showing the relative enrichment of immune cell types across different tissue regions inferred using ssGSEA scores. FAP^+^ fibroblasts and macrophages are predominantly enriched in tumor-associated stromal zones. **(C)** Spatial expression maps of key marker genes (CD3D, CD4, GZMA, GNLY, CD68, CD163, SPP1, and FAP), indicating strong spatial overlap between FAP, SPP1, and macrophage markers in tumor areas. Insets highlight regions of co-localization. **(D)** Correlation analysis of FAP^+^ fibroblast and macrophage scores in TCGA PRAD bulk RNA-seq dataset using ssGSEA; a strong positive correlation is observed (R = 0.69, p < 1e-60). **(E)** Association between FAP expression and immune cell infiltration levels in TCGA PRAD cohort assessed via TIMER. FAP expression positively correlates with macrophage infiltration, but not with T or B cells.

Recent years, effect of the intercellular communications between CAFs and TAMs on tumor progression has gained more and more comprehensive attention (34–36). Therefore, we propose a hypothesis that the anomalous interactions between FAP+ fibroblasts and TAMs could significantly contribute to the aberrant biological activities of FAP+ fibroblasts within the PCa TME. Herein, we utilized ST data to evaluate the spatial distribution of FAP+ fibroblasts and immune cells within the PCa TME. By analyzing hematoxylin and eosin staining slices and differentially expressed genes of each cell population after clustering, we categorized the ST spots into four primary cell populations: tumor cells, fibroblasts, epithelial cells, and smooth muscle cells (**Figure 2A**). We used ssGSEA algorithm to evaluate the infiltration of FAP+ fibroblasts and various kinds of immune cells with the genelist (**Supplementary Table S4**) in ST data (**Figure 2B**). Coexistence of FAP+ fibroblasts and macrophages were found in the area of fibroblasts while part of cytotoxic immune cells like CD8 T cells and NK cells were also enriched in that area (**Supplementary Table S5**). Further, we noticed that regions with high expression of FAP gene highly overlapped with regions with high expression of TAMs characteristic genes (CD68 and CD163), but there was no significant correlation with regions with high expression levels of T cells and B cells cell markers (**Figure 2C**). To further validate the association between FAP+ fibroblasts and TAMs, we utilized bulk RNA sequencing datasets (TCGA cohorts PRAD). ssGSEA was employed to calculate the expression levels of FAP+fibroblasts and macrophages within the TCGA dataset following correlation analysis. We found that the expression of FAP+ fibroblasts enhanced with the increased expression of macrophages (**Figure 2D**). Finally, by means of online tools TIMER, we investigated the relation between FAP expression and immune cell infiltration. We found that FAP had the strongest correlation with macrophages among all types of immune cell (**Figure 2E**).

In conclusion, these findings emphasize the presence of extensive intercellular connection between FAP+ fibroblasts and TAMs, potentially having the function of promoting PCa progression within the TME.

### Cellular crosstalk between FAP+fibroblasts and SPP1+ macrophages are related to poor outcomes in patients with PCa

**Figure 3:**
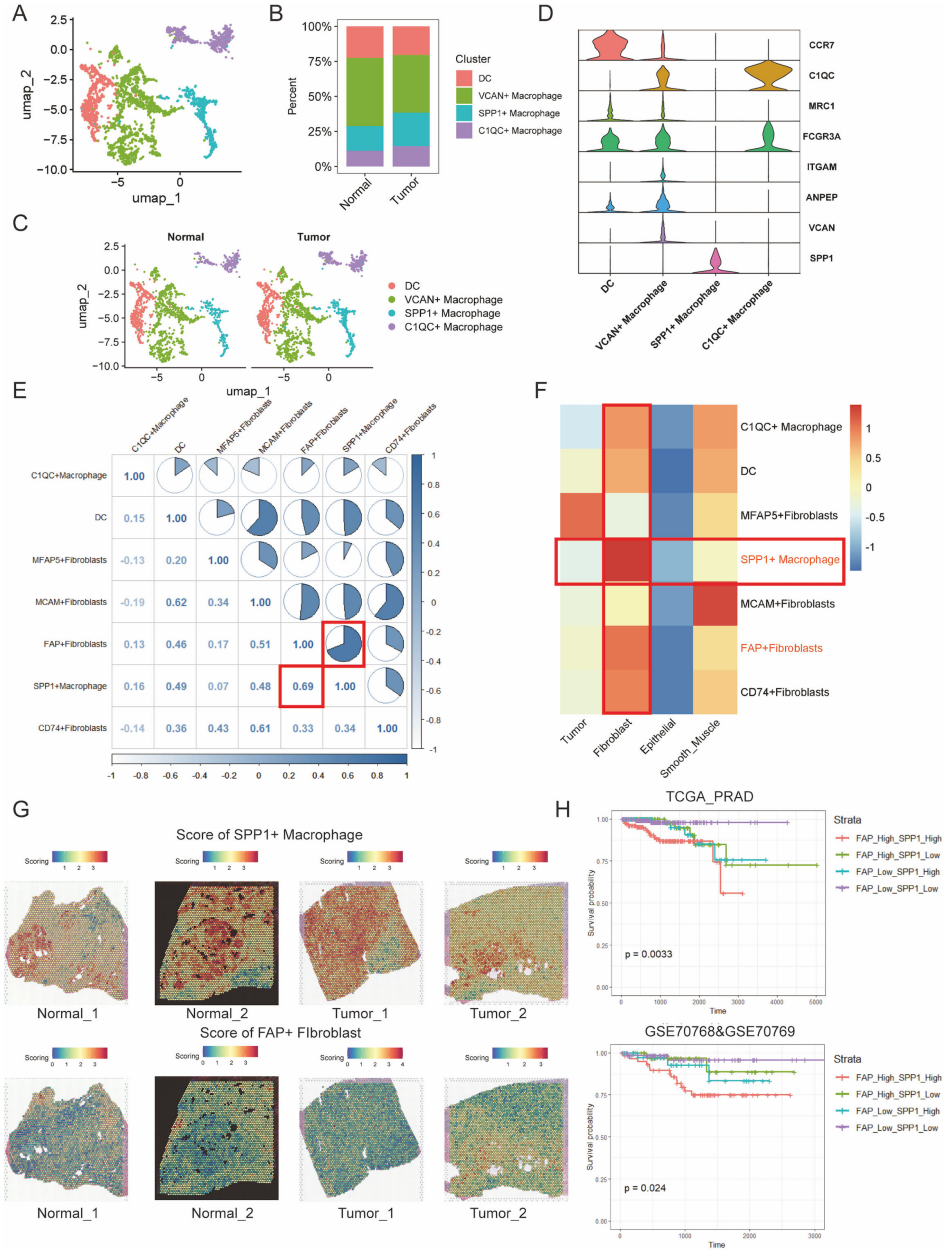
Cell to cell interactions of FAP + fibroblasts and SPP1 + macrophages are associated with poor PCa prognosis. **(A)** UMAP clustering of immune cell types in prostate cancer tissues, showing distinct populations of macrophages including DCs, VCAN^+^ macrophages, SPP1^+^ macrophages, and C10QC^+^ macrophages. **(B)** Percentage distribution of immune cell clusters in normal and tumor samples. **(C)** UMAP visualization of macrophage clusters in both normal and tumor tissues, highlighting differences in immune cell profiles. **(D)** Violin plots showing the expression of key markers for macrophage subsets (e.g., CCR7, C10QC, MRC1) in the various immune cell clusters. **(E)** Correlation analysis of marker gene scores between fibroblast and macrophage subtypes. A strong positive correlation is observed between FAP^+^ fibroblasts and SPP1^+^ macrophages (R = 0.69). **(F)** Heatmap showing the correlation matrix of FAP^+^ fibroblasts, SPP1^+^ macrophages, and other fibroblast subtypes, emphasizing the strong relationship between SPP1^+^ macrophages and FAP^+^ fibroblasts. **(G)** Spatial mapping of SPP1^+^ macrophage and FAP^+^ fibroblast scores across normal and tumor tissue sections, visualized using the ssGSEA algorithm. High co-localization of these cell types is observed in tumor areas. **(H)** Kaplan-Meier survival curves based on the combined infiltration levels of FAP^+^ fibroblasts and SPP1^+^ macrophages in the TCGA PRAD cohort and GSE70768/GSE70769 cohorts. Patients with high levels of both FAP^+^ fibroblasts and SPP1^+^ macrophages show significantly poorer survival outcomes.

TAMs are predominant tumor-infiltrating immune cells, known for their immune-suppressive and tumor-promoting functions (37). As mentioned above, we have demonstrated the close distance between FAP+ fibroblasts and TAMs in tumor region of PCa. However, it is unclear that which subtype of macrophages communicate with FAP+ fibroblasts. Thus, we further subjected TAMs to dimensional reduction and clustering analysis and divided them into four subgroups (**Supplementary Figure S7**). According to our analysis, dendritic cells (DCs) were characterized for their high expression of CCR7 (38). Similarly, macrophages featured by high levels of VCAN, SPP1 and C1QC were defined as VCAN+ macrophages (39), SPP1+macrophages and C1QC+macrophages respectively (**Figures 3A-D**). The results are consistent with previous studies on TAMs.

**Figure 4:**
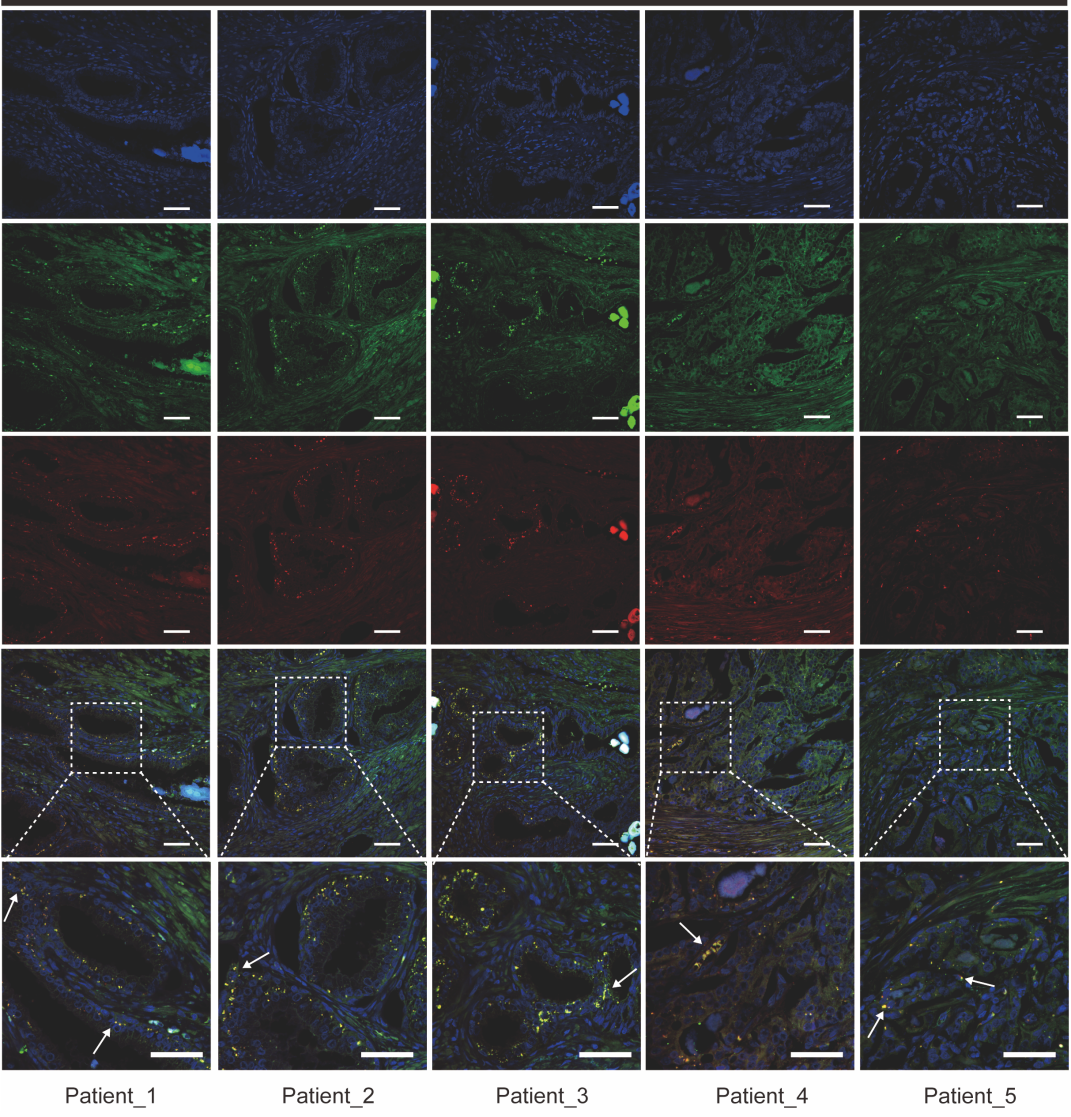
Immunofluorescence staining reveals co-localization of FAP^+^ fibroblasts and SPP1^+^ macrophages in prostate cancer tissue sections from five patients. Representative images showing the expression of FAP (green), SPP1 (red), and DAPI (blue) in prostate cancer tissue samples from five patients (Patient 1–5). Co-localization of FAP^+^ fibroblasts and SPP1^+^ macrophages is indicated by yellow signals (arrows) in the merged images. Higher magnification views of selected regions are shown in the inset panels. Scale bars = 50 µm (upper and middle panels), 200 µm (bottom panels).

Then, we obtained gene scoring signatures for different subpopulations of CAFs and TAMs through marker genes from scRNA-seq of PCa. Further, we scored the gene expression data in the TCGA dataset using the ssGSEA method. Subsequently, we determined the specific TAMs subtype interacting with FAP+ fibroblasts through correlation analysis. And the analysis revealed a significant positive correlation between FAP+ fibroblasts and SPP1+ macrophages, with the highest correlation coefficient observed (**Figures 3E, F**). Additionally, ST data was also analyzed to validate our findings in the TCGA dataset. Scores for individual cell/spot were calculated using five different algorithms including AUCell, UCell, singscore, ssGSEA, and AddModuleScore. The average score was calculated and visualized on ST sections. The findings suggested that FAP+ fibroblasts and SPP1+ macrophages were coexisted in the fibroblast regions (**Figure 3G**). In contrast, in normal tissue, there were few SPP1+ macrophages surrounding FAP+ fibroblasts, indicating that the interactivity between them primarily occurs within tumor tissues rather than in normal tissues. These findings were also demonstrated by immunofluorescence of FAP and SPP1 in PCa (**Figure 4**).

The protein encoded by FAP gene is a type of serine protease known as a membrane-associated gelatinase, which is specifically expressed in fibroblasts (37). The remodulation of the TME by FAP+ fibroblasts through reshaping the extracellular matrix have already been reported, and the overexpression of FAP+ fibroblasts indicates poor prognosis in various cancers (38). SPP1+ macrophages are one type of TAMs that may be induced by the hypoxic microenvironment of tumors with immunosuppressive property. The interactions between SPP1+ macrophages and FAP+ fibroblasts could be a prospective point for anti-tumor progression and metastasis (39–41). Clinical value of SPP1+ macrophages infiltration was then assessed in two independent PCa cohorts. It was noticed that low level of FAP+ fibroblasts and SPP1+ macrophages infiltration in patients tend to be with the best prognosis. In contrast, high infiltration level of FAP+ fibroblasts and SPP1+ macrophages lead to the poorest outcome in the two cohorts (**Figure 3H**).

To account for confounding factors such as age and pathological staging, we employed the Cox regression method to analyze patients’ prognoses. The results indicated that lower infiltration level of FAP+ fibroblasts and SPP1+ macrophages represents better outcomes in patients with PCa(**Supplementary Figure S9**). This finding supports our hypothesis that abnormal signaling interactions between FAP+ fibroblasts and SPP1+ macrophages lead to poor prognoses in PCa patients. Additionally, we conducted further analyses to explore the specific intercellular communication pathways between FAP+ fibroblasts and SPP1+ macrophages.

### Intercellular communication analysis reveals tumor−specific signaling pathways between CAFs and TAMs in PCa

**Figure 5:**
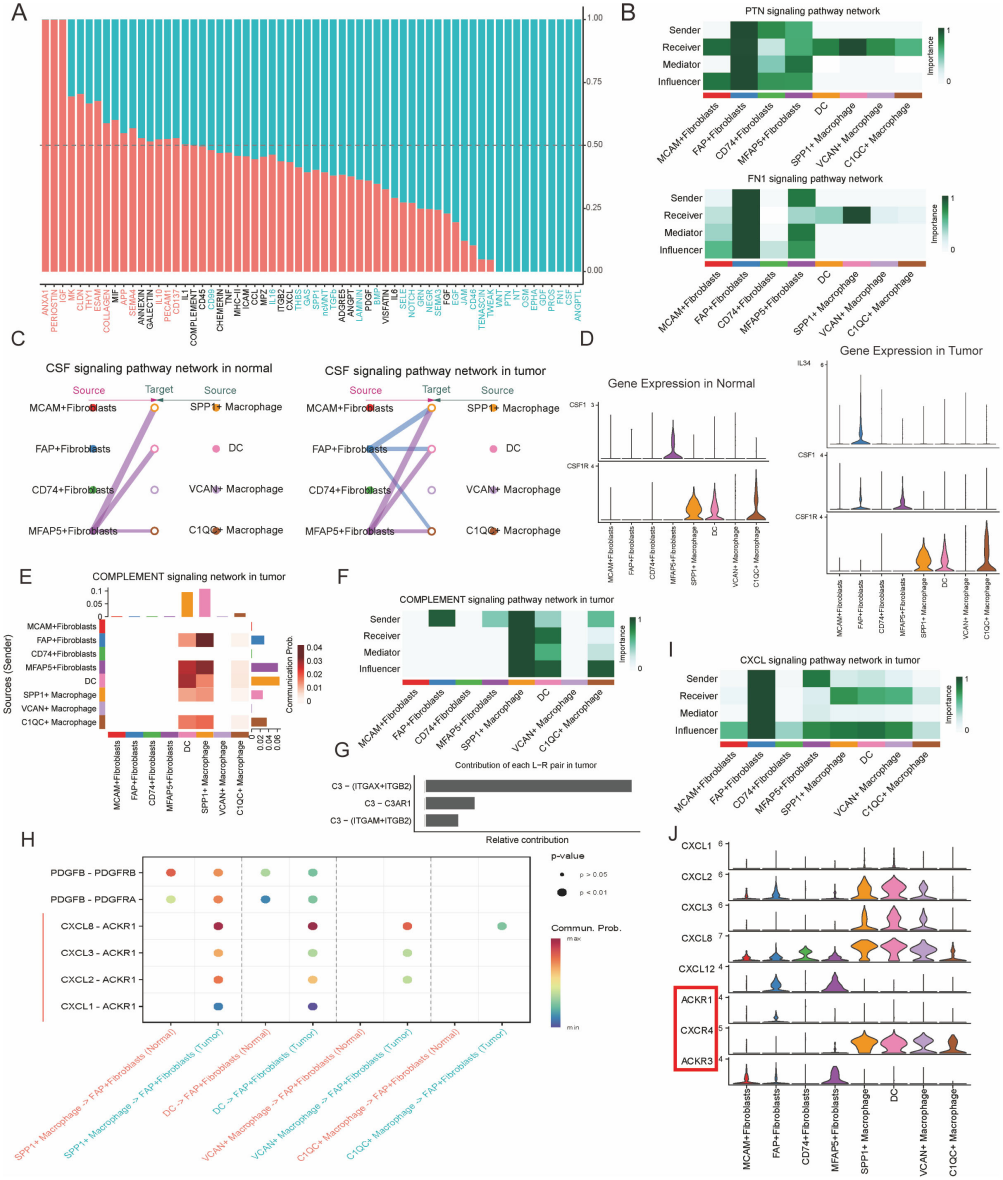
FAP+ fibroblasts modulate the phenotype of SPP1+ macrophages through the CSF/CSF1R signal pathway. **(A)** Bar plot of specific signaling pathways between fibroblasts and myeloid cells inferred by cellchat, with red indicating increased signals in normal tissue and blue indicating increased interactions in tumor tissue. **(B)** Heatmaps illustrating the interactions in the PTN and FN1 signaling pathway networks within the tumor microenvironment, highlighting key senders, receivers, and mediators among immune and stromal cells. **(C)** CSF signaling pathway networks in normal and tumor prostate tissues. The diagram shows the interaction between different fibroblast subtypes and macrophages, with FAP^+^ fibroblasts and SPP1^+^ macrophages highlighted in the tumor microenvironment. **(D)** Gene expression of CSF1 and related markers (CSF1R, C3) in normal and tumor tissues. Violin plots show distinct expression patterns of these genes across different immune and stromal cell populations. **(E)** Contribution of different signaling pathways in the complement network within the tumor, demonstrating the involvement of key fibroblasts and macrophage subtypes. **(F)** Sender-receiver interaction matrix for the complement signaling pathway, showing the relative roles of various cell types in the signaling network. **(G)** Contribution of individual signaling pairs in the complement pathway network, highlighting the key interactions between C3 and its receptors in the tumor microenvironment. **(H)** Dot plot shows the expression of L-R pairs of CXCL and EGF signaling pathway between fibroblasts and myeloid cells in normal (red color) or tumor (green color) tissues. **(I)** CXCL signaling pathway network in tumor tissue, focusing on the communication between macrophages and fibroblasts. **(J)** Violin plots of gene expression for CXCL ligands and their corresponding receptors, including ACKR1, CXCR4, and ACKR4, across various tumor and immune cell types, highlighting their roles in the tumor microenvironment.

Previous studies have indicated that there are extensive reciprocal intercellular communications between CAFs and TAMs. These interactions make a great contribution to the biological behavior of tumor development, progression, and metastasis. To investigate the specific signaling pathways through which intercellular interactions among these cell clusters lead to a poorer prognosis in patients with PCa, we analyzed the intercellular communication networks between CAFs and TAMs in both normal and tumor tissues (**Supplementary Tables S10**-**S12**). We found that in tumor tissues, FAP+ and MFAP5+ fibroblasts have most communication pathways with TAMs (**Supplementary Figure S8**), suggesting that FAP+ and MFAP5+ fibroblasts play an important regulatory role within the TME. Compared to the intercellular interactions in normal tissues, we noted several tumor-promoting signaling pathways specificly found in PCa tissues, including ANGPTL, FN1, GDF, CSF, PTN, and LAMININ signaling pathways (**Figure 5A**). Analysis of the communication patterns of CAFs and TAMs also showed a coordinated tumor-promoting signaling communication model between FAP+ and MFAP5+ fibroblasts (**Figures 5B, C**). Previous literature has also confirmed that these two types of fibroblasts are important driver subpopulations of stromal cells in tumorigenesis (42, 43). Hence, we infer that there is a coordinated cellular communication pathway between FAP+ fibroblasts and MFAP5+ fibroblasts, jointly driving the occurrence, development, and metastasis of tumors.

In conclusion, our research highlights the complex intercellular communications between FAP+ fibroblasts and TAMs that exacerbate PCa malignancy. Targeting these tumor-specific intercellular communication pathways offers a promising therapeutic strategy for treating PCa.

TAMs are an important component of innate immune cells within the tumor microenvironment (44). Monocytes in the blood are recruited into tumor tissues, where they are induced to differentiate into TAMs (45, 46). Specific cytokines and growth factors are indispensable for the proliferation and differentiation of TAMs, comprising of colony stimulating factor-1 (CSF1), interleukins and so on. Through Cellchat analysis, we found that the CSF pathway was one of the key points in the regulatory network of cell interactions between CAFs and TAMs in the TME. We noticed that SPP1+ macrophages primarily receive CSF signals secreted by MFAP5+ fibroblasts in normal prostate tissues. However, in PCa, CSF signals from MFAP5+ and FAP+ fibroblasts synergistically act on SPP1+ macrophages and C1QC+ macrophages, indicating a significant enhancement of the CSF signaling pathway in PCa (**Figure 5C**).

Furthermore, when exploring the ligand and receptor expression of the CSF signaling pathways, it showed that in normal tissues only MFAP5+ fibroblasts secrete CSF1, which acting on the CSF1R of SPP1+ and C1QC+ macrophages. However, in PCa tissues, CSF factor and IL34 secreted by FAP+ fibroblasts together act on the CSF1R of SPP1+ and C1QC+ macrophages (**Figure 5D**), suggesting that in PCa, the interaction between FAP+ fibroblasts and SPP1+ macrophages through the CSF pathway are significantly enhanced and altered. Functional enrichment analysis indicates that SPP1+ macrophages could promote angiogenesis in tumor tissues. Previous studies showed angiogenesis of tumor tissue is one of the risk of tumor metastasis (47, 48). What’s more, our research suggests that the enhancement of the CSF1-CSF1R and IL34-CSF1R signaling pathways might be an important reason for the enhanced angiogenic capabilities of SPP1+ macrophages resulting in metastasis and poor prognosis in PCa patients with high infiltration levels of both FAP+ fibroblasts and SPP1+ macrophages. Therefore, CSF1 blockade may be an important potential target for interrupting this process and improving the prognosis of patients with PCa (49).

### Positive regulatory loop between FAP + fibroblasts and TAMs attributes to the progression of PCa

SPP1+macrophages are reported to be pro-tumorigenic TAMs with the function of complement activation, antigen processing and presentation and so on (50). They are believed to be induced and regulated by the metabolites, cytokines and other bioactive substances within the TME (51). However, the specific signaling pathways regulating SPP1+macrophages have not been fully classified. In our research, we found that FAP+ fibroblasts and SPP1+ macrophages communicate closely to each other.

We then explored whether FAP+ fibroblasts have the ability to regulate the immune responses of SPP1+macrophages. It was observed that FAP + fibroblasts were the unique source of complement C3, which interacts with the receptors ITGAM/ITGB2, ITGAX/ITGB2, and C3AR1 on SPP1+macrophages within the TME, thereby activating the immune response (**Figures 5E-G**). Hence, FAP+ fibroblasts might abnormally activate SPP1+macrophages through the complement pathway and attribute to the tumorigenesis, progress, and metastasis of PCa (30, 36, 52).

Besides the immune regulation functions of FAP+ fibroblasts, we also explored the cellular interactions dominated by TAMs. We found that C-X-C Motif Chemokine Ligand (CXCL) signaling was remarkably upregulated in TAMs compared with macrophages resident in normal prostate tissues. CXCL chemokines (CXCL1, CXCL2, CXCL3 and CXCL8) secreted by TAMs bind to the ACKR1 receptor specificly expressed on FAP+ fibroblasts (**Figures 5H-J**). Overexpression of CXCL signal pathway has been found to be one of the hinges on tumor growth, angiogenesis, invasion and metastasis, as well as drug resistance (53). Thus, the aberrant activation of ACKR1 receptor and its downstream signaling pathway in FAP+ fibroblasts could be a partial reason for their malignant action. In conclusion, FAP+ fibroblasts could motivate the proliferation and tumor angiogenesis characteristics SPP1+macrophages. Conversely, SPP1+ macrophages could also enhance the aggressive phenotypes of FAP+ fibroblasts by secreting chemokines including CXCL1, CXCL2, CXCL3, CXCL8 and so on. These reciprocal intercellular communications form a positive regulatory loop attributing to the malignant TME in patients with PCa.

## Discussion

PCa is one of the most common cancers of the male urinary system with high and long-term survival (54). Although carefully monitoring are enough for patients with low-risk and localized primary PCa (1), the mortality rate for patients with advanced PCa remains high, necessitating a multidisciplinary treatment including surgery, radiation therapy, and hormone therapy (55). It was reported that approximately 15% of patients with PCa are diagnosed with advanced PCa (56). However, the specific mechanisms driving immune escape and tumor progression in advanced PCa have not yet been elucidated.

**Figure 6:**
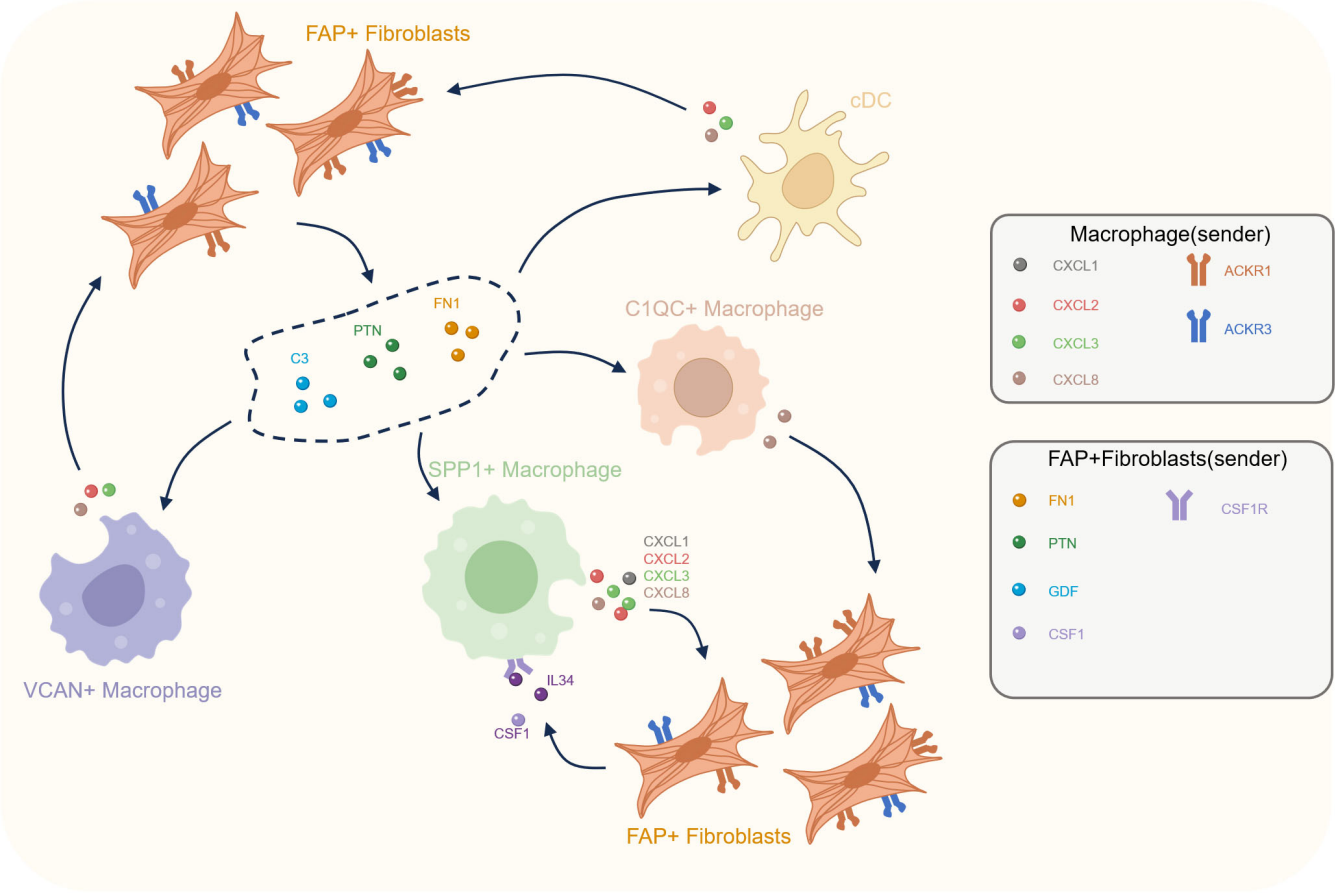
Cellular crosstalk between FAP^+^ fibroblasts and macrophages in the prostate cancer tumor microenvironment. This diagram illustrates the reciprocal interactions between various macrophage subtypes (C1QC^+^, SPP1^+^, VCAN^+^) and FAP^+^ fibroblasts within the tumor microenvironment. FAP^+^ fibroblasts secrete key signaling molecules, including FN1, PTN, C3, CSF1, and IL34, that influence macrophage activation and recruitment. In turn, macrophages (especially SPP1^+^ macrophages) release cytokines such as CXCL ligands, which act on FAP^+^ fibroblasts to further modulate the TME.

Recent years, with the emergence and progress of scRNA-seq and ST, the details of TME could be further detected and explored (57). Intercellular communications networks are an important part of the TME and have been reported to be associated with the malignancy property of cancers (58, 59). CAFs and TAMs are the most two common types of non-tumor cells in solid cancers with extensive intercellular communications and transformation (35, 60, 61). It is believed that an in-depth identification of intercellular communications between CAFs and TAMs could help us to find new novel therapeutic targets for PCa. Therefore, we integrated bulk RNA-seq, scRNA-seq and ST to explore the detailed intercellular communications between CAFs and TAMs with IF and IHC experiments to validate our findings (**Figure 6**).

The diversity of CAFs significantly influences the proliferation, differentiation and function of tumor infiltrating immune cells (62, 63). Recent studies have identified several novel fibroblast subclusters with different gene expression and functions such as ACTA2+ myofibroblasts and FAP+ fibroblasts (64). These investigations have highlighted that CAFs are activated fibroblasts typically expressing markers like FAP, FSP, and αSMA, which are important in promoting tumor growth and resulting in chemotherapy resistance (31). FAP+ fibroblasts are involved in multiple biological processes including tissue repair, fibrosis, and extracellular matrix degradation with the expression of collagenase and dipeptidyl peptidase (65, 66). It is reported that both cancer and stromal cells undergo hypoxic necrosis rapidly in FAP depleted transgenic mice models of Lewis lung carcinoma or pancreatic ductal adenocarcinoma (67). However, up to now, there has not been research investigating the effect of FAP+ fibroblasts on the TME of PCa. Therefore, our research focused on the pivotal role of newly identified FAP+ fibroblasts in PCa, investigating their biological characteristics and impact on the TME. It is found that FAP+ fibroblasts predominantly reside in PCa tissues rather than normal PCa tissues. What’s more, patients with higher level of infiltration of FAP+ fibroblasts have poorer prognoses compared to those with lower level of infiltration, indicating their pro-tumoric effects.

Increasing evidence indicates that fibroblasts are able to communicate with different immune cells by secreting cytokines, chemokines and other biomolecules, which attributes to the formation of an immunosuppressive microenvironment and facilitates tumor immune tolerance (68–70). Kumar V et al. found that FAP+ fibroblasts were associated with the infiltration of immune cells in gastric cancer (71). In our study, we explored the relationship between FAP+ fibroblasts and multiple tumor infiltrating immune cell types in PCa. To ensure the validity of our findings, we integrated multi-omics data, including bulk RNA-seq, scRNA-seq and ST. For the first time in PCa, we identified a significant correlation between FAP+ fibroblasts and TAMs.

These findings underscore that the intercellular communications between FAP+ fibroblasts and SPP1+ macrophages are crucial in influencing the malignant phenotype and leading to poor prognosis in patients with PCa. Immune cells have an important impact on tumor prognosis(PMID:38101174). Recent studies have further demonstrated that cellular interactions within the TME are closely linked to immunotherapy outcomes. For instance, Ye et al. proposed a multi-omics-based model (iMLGAM) showing that immune-active environments are associated with improved response to checkpoint blockade (72). Another pan-cancer study by the same group identified a plasma cell signature predictive of immunotherapy efficacy, reinforcing the value of immune infiltration as a prognostic indicator (73). These findings support our conclusion that the FAP+ fibroblast–SPP1+ macrophage axis fosters an immunosuppressive microenvironment contributing to poor prognosis in PCa.

Regarding the heterogeneity of TAMs, further research is needed to identify the specific subtype of TAMs that interact with FAP+ fibroblasts. Through analysis of multi-omics sequencing datasets and validation of IHC and IF experiments, we demonstrated a close interaction between FAP+ fibroblasts and SPP1+ macrophages in PCa tissues. Previous research has revealed the immunosuppressive and angiogenesis properties of SPP1+ macrophages, which could lead to poorer clinical outcomes in PCa patients (74, 75). In our research, the survival analysis indicates that patients with high infiltration level of both FAP+ fibroblasts and SPP1+ macrophages have the poorest prognosis compared to those with lower infiltration levels of either FAP+ fibroblasts or SPP1+macrophages. IHC and IF experiments reveal that FAP+ fibroblasts could attach to SPP1+ macrophages in border stromal regions, prompting interactions with each other. These findings underscore that the intercellular communications between FAP+ fibroblasts and SPP1+ macrophages are crucial in influencing the malignant phenotype and leading to poor prognosis in patients with PCa.

Given the important role of FAP+ fibroblasts in modulating pro-tumoric signaling pathways within the PCa TME, we performed intercellular correlation analyses to uncover potential tumor-driven mechanisms. Our findings identify several tumor-specific interactions between FAP+ fibroblasts and TAMs, especially SPP1+ macrophages. Our analysis shows that FAP+ fibroblasts may activate SPP1+ macrophages through the CSF/CSF1R and IL34/CSF1R axis, and induce an immunosuppressive microenvironment via GDF15/TGFBR2 signaling (76). High infiltration level of FAP+ fibroblasts infiltration also contributes to the formation of a desmoplastic microenvironment through laminin pathways and other intercellular signals creating conditions unfavorable for anti-tumor immunity. Moreover, coordinated signaling between FAP+ fibroblasts and MFAP5+ fibroblasts further exacerbate the invasive characteristics of the PCa microenvironment. Reciprocally, activated TAMs also help sustain the activation of FAP+ fibroblasts in a positive feedback loop via CXCL signaling pathway. These communication pathways provide potential new targets for future PCa therapies.

There are still some limitations in our study. For example, the sample size we analyzed is relatively small and the biological characteristics of FAP+ fibroblasts are investigated only in PCa. Therefore, it still remains uncertain whether FAP+ fibroblasts are a conserved cell type across various cancer types. Previous research has revealed high levels of FAP expression is associated with poor prognosis in patients with colon and bladder cancers. However, further investigations are still necessary to clarify the role and properties of FAP+ fibroblasts across different kinds of cancers.

## Conclusions

In this study, we utilized multi-omics datasets of bulk RNA-seq, scRNA-seq and ST to investigate the detailed landscape of the PCa microenvironment. Our findings provide an extensive insight into the function of FAP+ fibroblasts, particularly focusing on their interactions with SPP1+macrophages and other TAMs. This could promote the development of targeted treatment strategies to improve outcomes for patients with PCa.

## Data Availability

The original contributions presented in the study are included in the article/**Supplementary Material**. Further inquiries can be directed to the corresponding author.
