# Supplementary material for: Intercellular communication between FAP+ fibroblasts and SPP1+ macrophages in prostate cancer via multi-omics
Source: Front Immunol. 2025 May 14;16:1560998. doi: 10.3389/fimmu.2025.1560998 (PMC12116517; doi:10.3389/fimmu.2025.1560998)
Supplement: Supplementary file 2 [file DataSheet2.docx]

Figure Supplyment


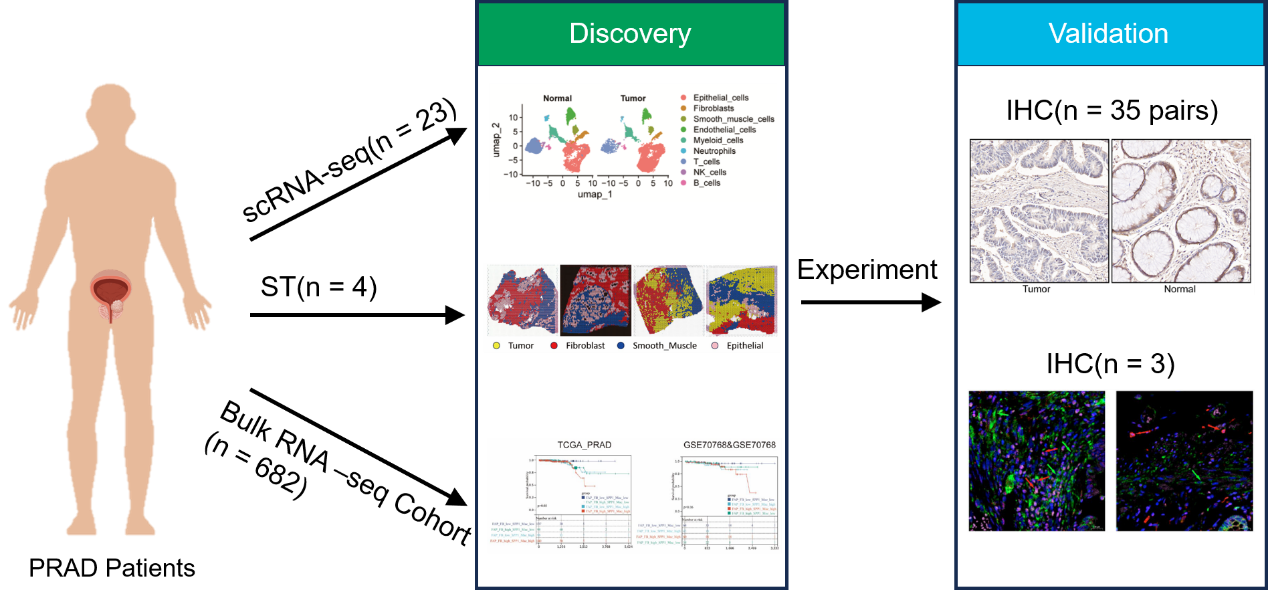


Fig. S1. Scheme illustrating the design of this study.


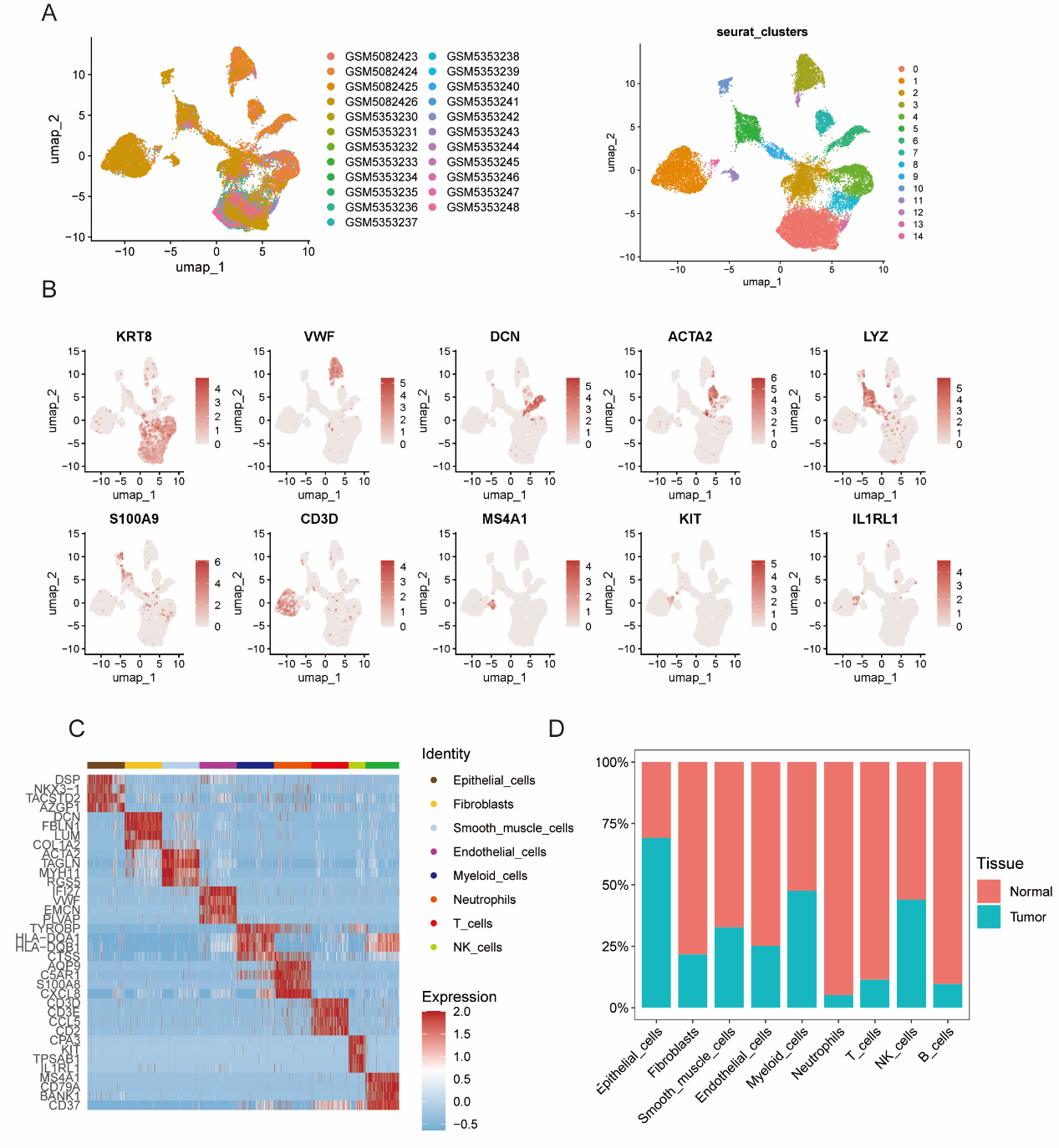


Fig. S2. Construction of scRNA-seq atlas in PCa tissues. (A) Integration and clustering analysis of the samples. (B) CellMarkers of different cell clusters in PCa tissues. (C) Heatmap exhibiting the five top high expressed genes in each cell type. (D) Bar plot showing the change of proportion of each kind of cells in normal prostate tissue and PCa.


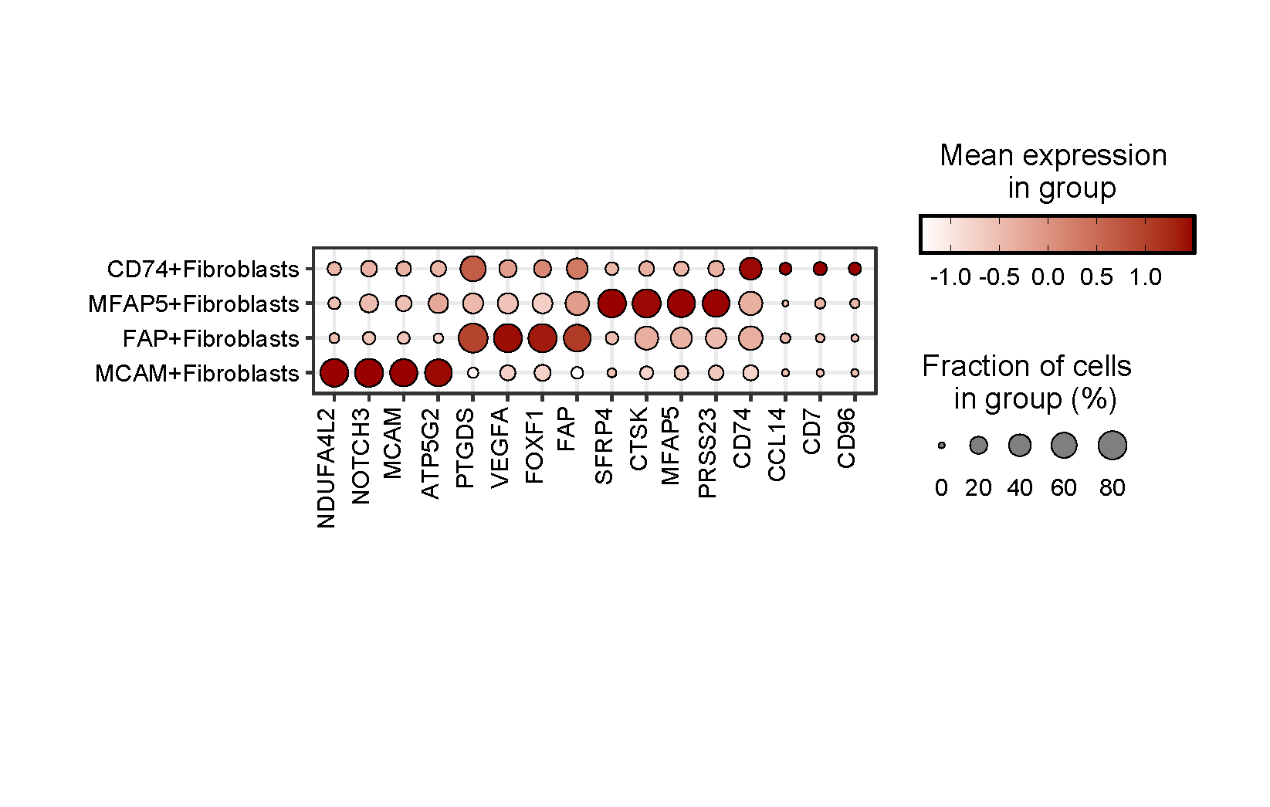


Fig. S3. The dotplot shows the marker genes of each fibroblast subcluster identified in scRNA-seq.


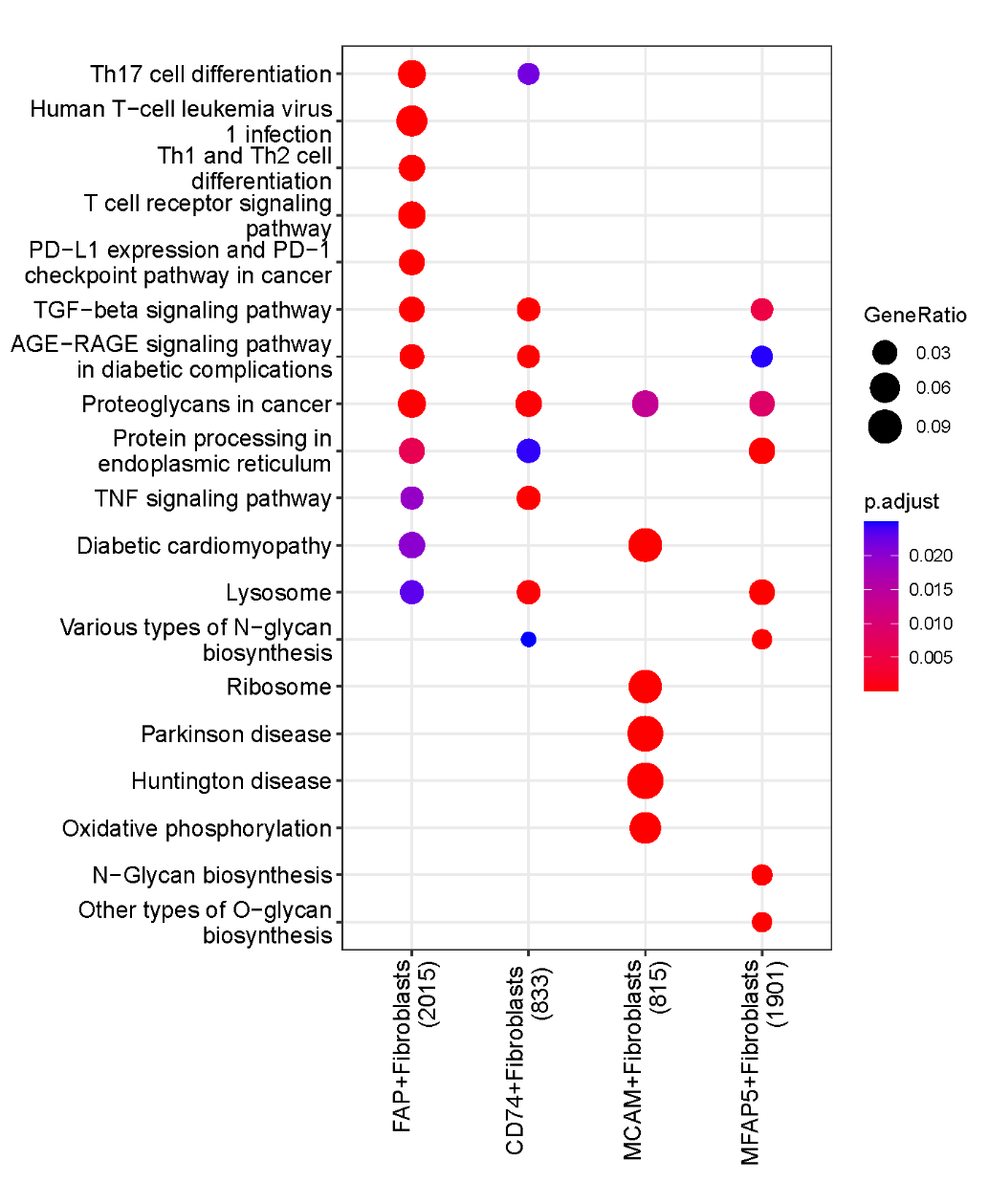


Fig. S4. GO enrichment analysis on four fibroblast subtypes identified in scRNA-seq.


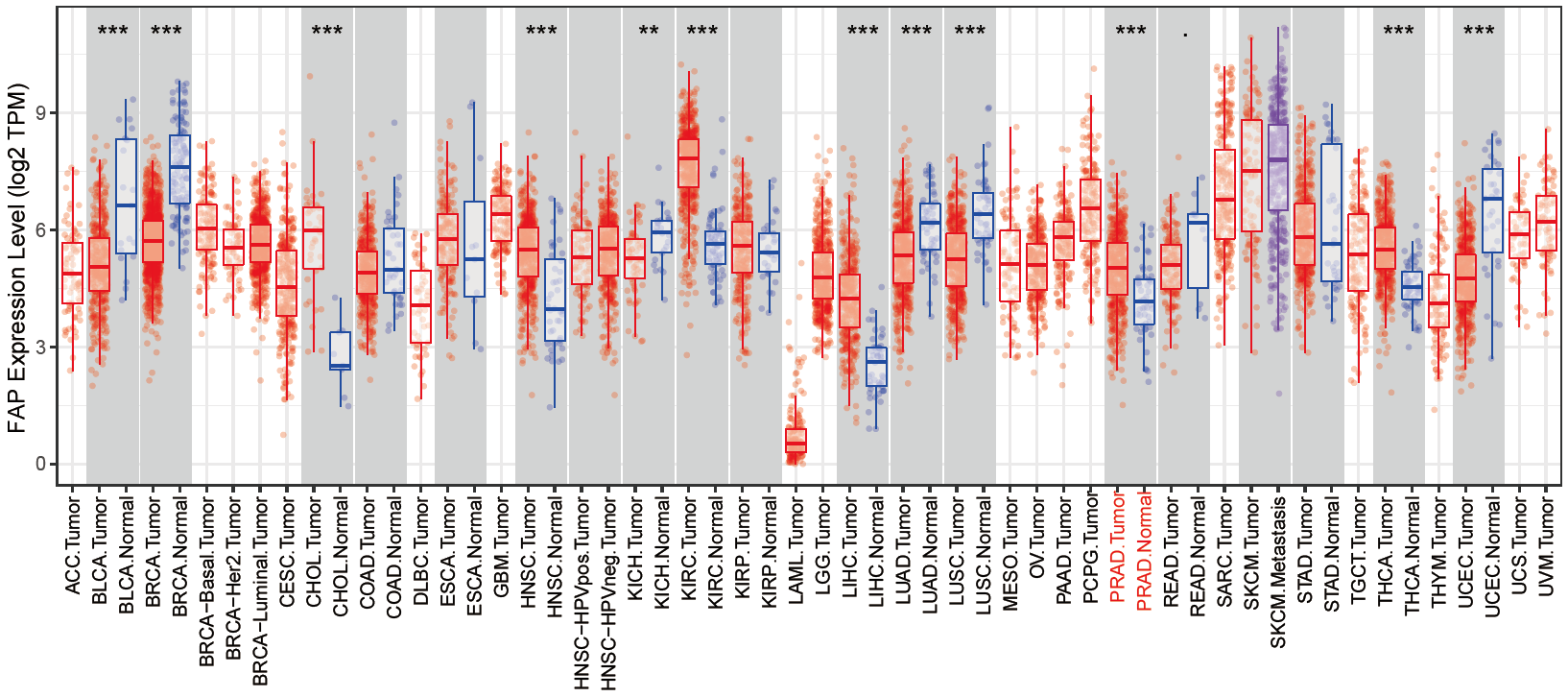


Fig. S5. Pan cancer analysis reveals that FAP is up-regulated in PCa tissues than in normal prostate tissues.


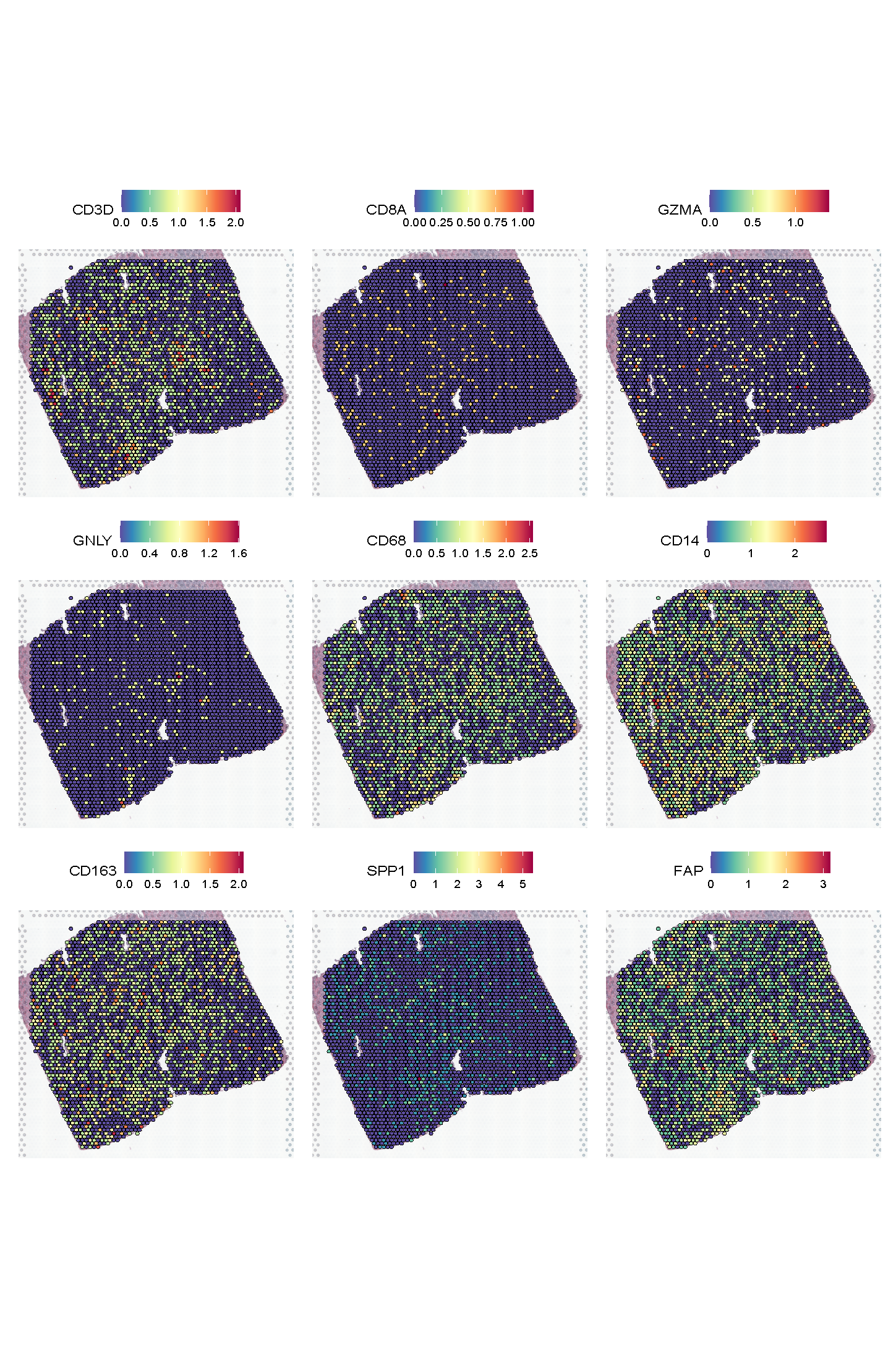


Fig. S6. Gene expression of CD3D, CD8A, CD19, MS4A1, GZMA, GNLY, CD68, CD163 as well as MFAP5+fibroblasts (top 20 DEGs identified in scRNA-seq) in spatial organization.


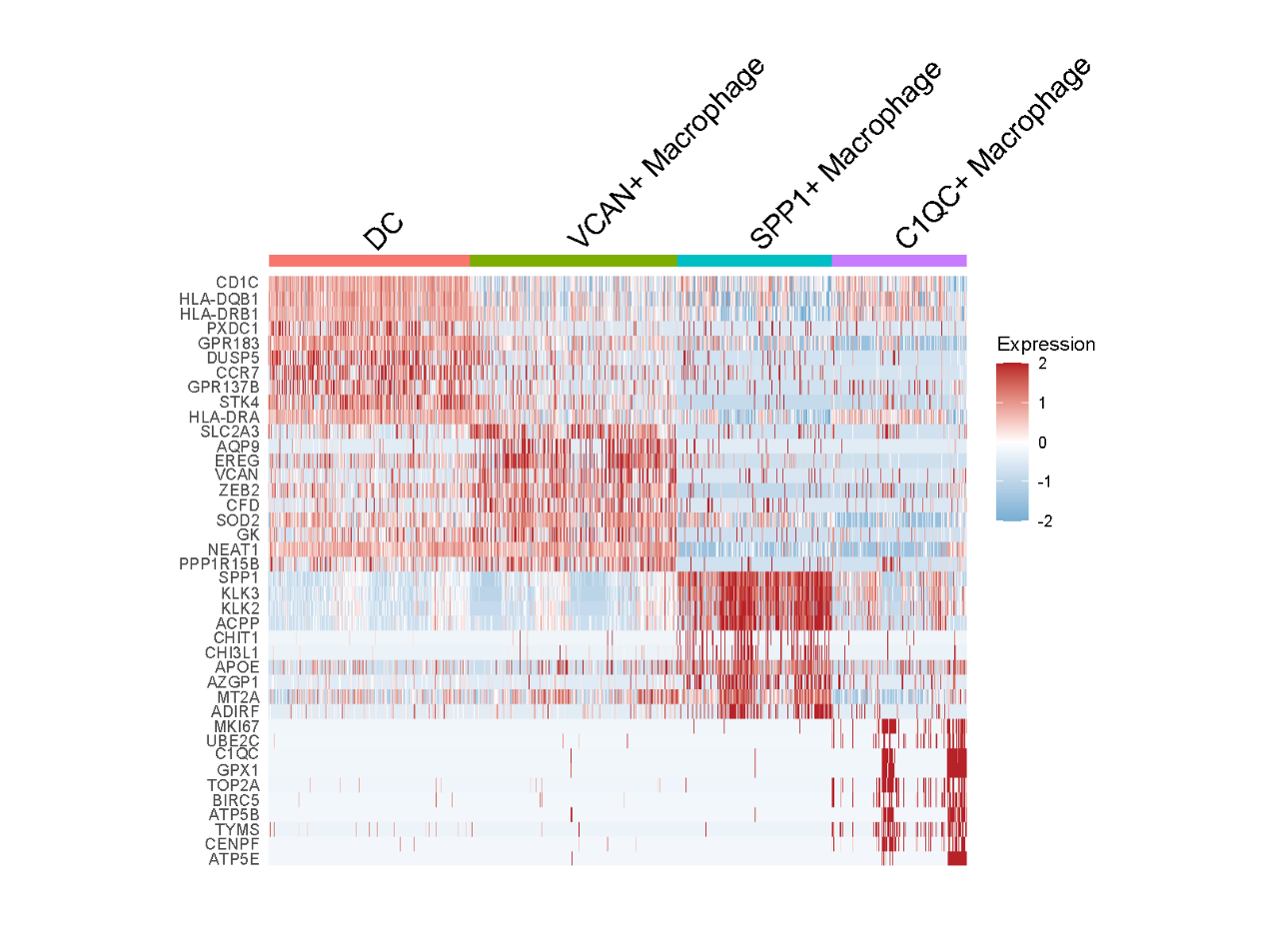


Fig. S7. Heatmap visualizes the top 10 DEGs of TAMs in scRNA-seq of patients with PCa.


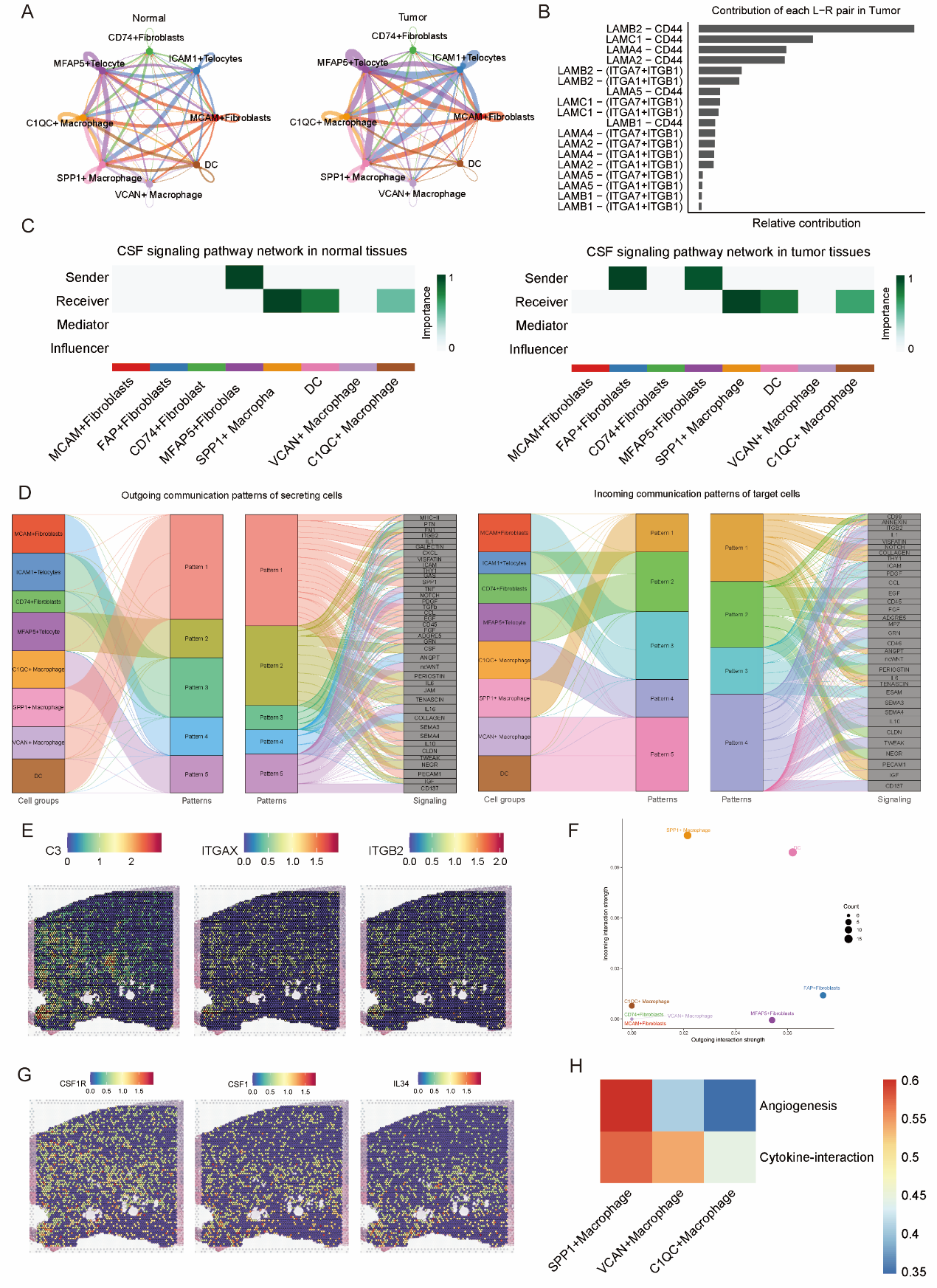


Fig. S8. Construction of intercellular communication network in PCa tissues. (A) The circle plots show the overview intensity of cellular communication between fibroblasts and myeloid cells in normal and tumor tissues respectively, arrow width represents the communication intensity. (B) Bar plot shows the relative contribution of each L-R pair to the overall LAMININ signaling network within the tumor tissue. (C) Heatmap shows dominant senders, receivers, mediators and influencers in CSF signals of tumor inferred by network centrality score. (D) The outgoing and incoming signaling patterns of certain cells in PCa tissue visualized by alluvial plot. (E) Spatial transcriptomics visualize the expression of C3, ITGAX and ITGB2. (F) Dot plot shows an increase in signal targeting SPP1 + macrophages by FAP + fibroblasts. (G) Spatial images localize the expression of CSF and CSF1R which suggest that the co-localization of CSF and CSF1R in PCa slices. (H) Heatmap showing angiogenesis and cytokine-interaction function scores of three identified tumor-associated macrophage subtypes.s


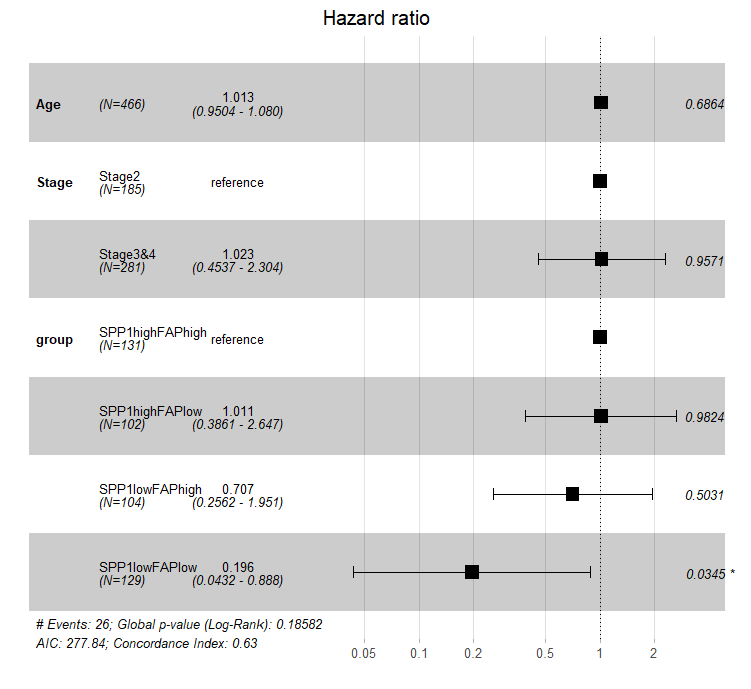


Fig. S9. Cox proportional hazard regression model established by adjusting proportion of FAP+fibroblasts_SPP1+macropahges, pathologic stage, age and gender in TCGA-PRAD cohort to identify independent risk factors of PFI.
